# Histological validation of calcifications in the human hippocampus as seen on computed tomography

**DOI:** 10.1371/journal.pone.0197073

**Published:** 2018-05-11

**Authors:** Melissa E. M. Peters, Remko Kockelkoren, Esther J. M. de Brouwer, Huiberdina L. Koek, Ronald L. A. W. Bleys, Willem P. Th. M. Mali, Jeroen Hendrikse, Annemieke M. Rozemuller, Pim A. de Jong

**Affiliations:** 1 Department of Radiology, University Medical Center Utrecht, Utrecht, The Netherlands; 2 Department of Geriatrics, University Medical Center Utrecht, Utrecht, The Netherlands; 3 Department of Anatomy, University Medical Center Utrecht, Utrecht, The Netherlands; 4 Department of Pathology, University Medical Center Utrecht, Utrecht, The Netherlands; University of Modena and Reggio Emilia, ITALY

## Abstract

**Background:**

Calcifications within the hippocampus were recently described for the first time on computed tomography (CT). These calcifications appeared in patients older than 50 years, the prevalence increases with age and they may be associated with cognitive decline. The aim of this study was to determine the histological basis (the presence, severity and location) of these CT-detected hippocampal calcifications of post-mortem brains.

**Methods:**

CT scans of seven post-mortem brains were scored for the presence and severity (mild, moderate, severe) of hippocampal calcification. After this, samples from nine hippocampi (bilateral in two brains, unilateral in five brains) were stained with hematoxylin and eosin (HE) to indicate the cytoarchitecture, with Elastica van Gieson to analyse the elastic connective tissue of the vessel walls and with von Kossa for detection of calcium.

**Results:**

In four brains (six hippocampi), calcifications were both found on CT and in corresponding histology. In three brains (three hippocampi), calcifications were absent on CT and corresponding histology. In histology, mild calcifications were located in the tail and severe calcifications involved the tail, body and sometimes the head of the hippocampus. The calcifications co-localised with precapillaries, capillaries and arteries of the molecular and granular layers of the dentate gyrus and the Cornu Ammonis 1.

**Conclusions:**

In this study, calcifications of the hippocampus as seen on CT scans were histologically located in vascular structures of the tail, body and head of the hippocampus.

## Introduction

Cerebrovascular disease is a major cause of morbidity and mortality worldwide. The main clinical diseases associated with intracranial vascular problems are stroke and dementia [1]. One of the phenomena commonly present in the intracranial vessels of cerebrovascular patients is the accumulation of calcium in the arterial wall. Large calcifications may cause occlusions and restrict blood flow to certain parts of the brain and calcifications may also stiffen arteries [2, 3]. Calcifications of different brain structures can be demonstrated with computed tomography (CT). The most common calcifications of the intracranial carotid artery [4, 3], the choroid plexus [5] and basal ganglia structures, like the globus pallidus and dentate nucleus [6, 7], have been observed on CT in human.

Calcification of the choroid plexus, which is responsible for the production of cerebrospinal fluid, increases in frequency at increasing age [5]. The calcification can involve the temporal horn, the floor of the body of the lateral ventricle, the roof of the third ventricle and the foramen of Monro [5]. These regions border the hippocampus, and calcifications of the hippocampus were therefore often misinterpreted as calcification of the choroid plexus [8, 9]. The evolution of CT scan techniques allowed for better quality of images and thinner CT slices, which can be read more easily in various viewing planes. On these scans, a difference between choroid plexus calcifications and adjacent hippocampal calcifications can be made.

Limited research has been performed on calcifications of the hippocampus. Chew *et al*. [9] were the first to describe hippocampal calcification on CT. They found that hippocampal calcification appears particularly in patients older than 50 years and that the prevalence increases with age [9].

A recent study by Kockelkoren *et al*. [8] investigated in a case-control study the presence of hippocampal calcification and the relationship with cognitive decline. In memory clinic patients calcifications of the hippocampus were more prevalent and associated with a lower cognitive functioning [8].

One histopathological study by Wegiel *et al*. [10] investigated calcifications located in the hippocampus in Alzheimer’s disease, Down syndrome, and control aging patients. They described the calcifications to be a manifestation of vascular disease, which they called vascular fibrosis and calcifications [10].

Currently, no evidence is available that the calcifications as observed on CT scans [8, 9] are indeed corresponding to vascular fibrosis and calcifications as found in the histopathological study [10]. For this reason, the aim of this study was to determine the histological basis of these CT-detected hippocampal calcifications in human post-mortem brains.

## Materials and methods

### Patients

For this study, we examined a total of seven brains. One brain was derived from a body, which was included in a donation program from the Department of Anatomy. Six brains were from patients who had an autopsy at the Department of Pathology. Local ethical committee approval was obtained for research on retained tissues after written informed consent given by the patients during life or their next of kin after death (Medical Ethics Committee of the University Medical Centre Utrecht 11-531/C). All brains were CT scanned post-mortem. Table 1 shows the available information of the seven patients about age, gender and relevant medical history.

**Table 1:** Patient information and relevant clinical history of the seven patients.

Patient number	Gender	Age (years)	Medical history	Number of hippocampi analysed
**1**	F	95	- Body donation to the Department of Anatomy without available medical history	2 hippocampi: head, body and tail
**2**	F	87	- Cerebrovascular Accident - Atrial fibrillation	2 hippocampi: head, body and tail
**3**	F	62	- Subarachnoid haemorrhage, intracranial aneurysm	1 hippocampus left
**4**	F	81	- Atrial fibrillation - Asthma - Hypertension - Sepsis and ischemia in leg	1 hippocampus right
**5**	M	60	- Stem cell transplantation - Human immunodeficiency virus - Acute myeloid leukemia - Sepsis and pulmonary infections - Secondary inflammation of pituitary	1 hippocampus left
**6**	M	71	- Sinus thrombosis - Heart attack - Polycythemia vera	1 hippocampus right
**7**	F	75	- Haemorrhage in the thalamus and pons - Atherosclerosis - Atrial fibrillation - Hypertension	1 hippocampus right

### CT examinations

**Fig 1:**
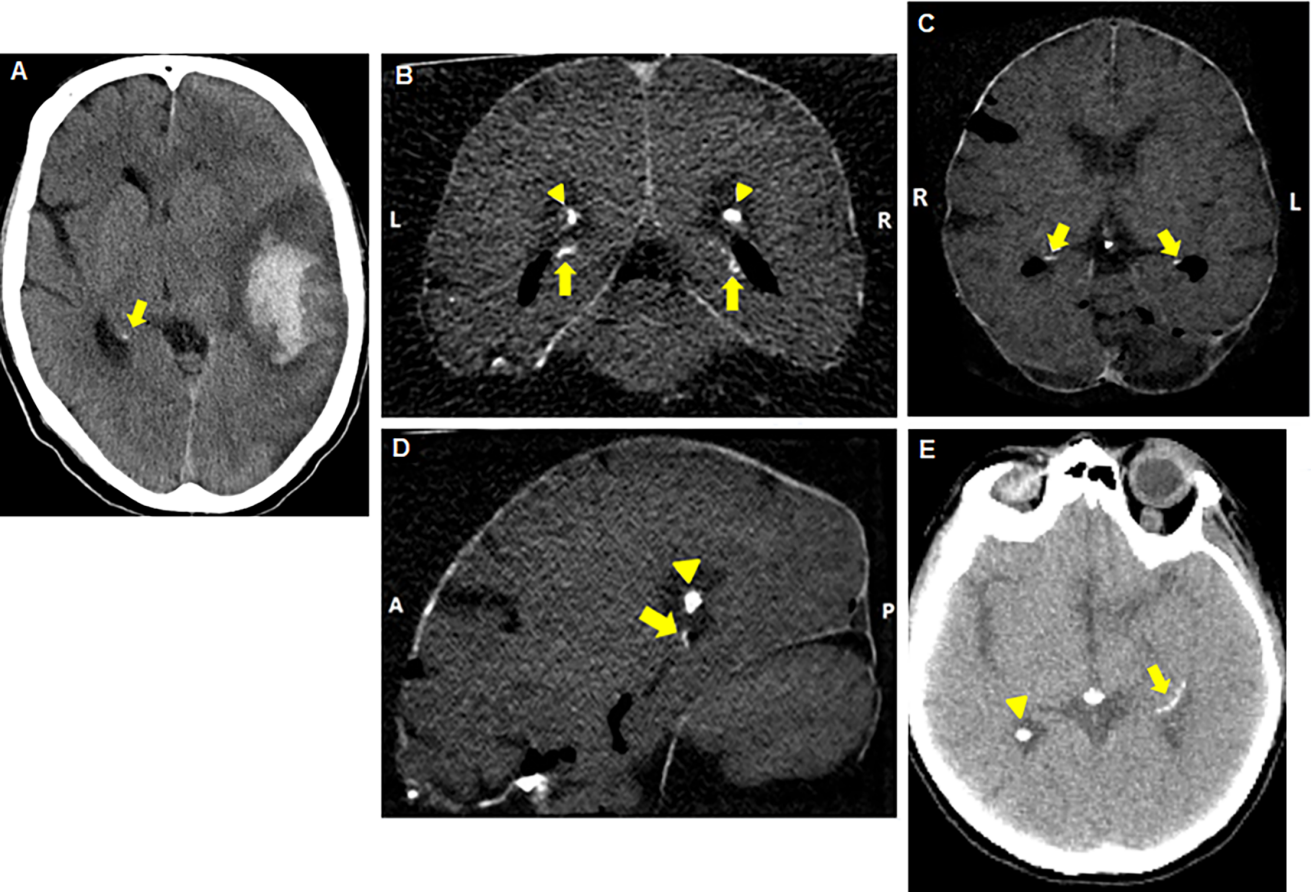
**Hippocampal calcifications on CT scans of patient numbers 4 (mild, A), 1 (moderate, B-D) and 3 (severe, E)**. (A) Axial reconstructed image with mild hippocampal calcification (one dot). (B) Coronal reconstructed image shows (moderate) bilateral hippocampal calcification (multiple dots), indicated by arrows. Choroid plexus calcification is indicated by arrowheads. (C) Axial reconstructed image with moderate bilateral hippocampal calcification marked with arrows. (D) Sagittal reconstructed image with moderate hippocampal calcification marked with an arrow and choroid plexus calcification marked with an arrowhead. (E) Axial reconstructed image shows severe hippocampal calcification (confluent) indicated by an arrow and calcification of the choroid plexus is indicated with an arrowhead.

The brain CT scans were made with a Philips Brilliance 64-slice or 256-slice CT scanner (Philips Healthcare, Best, The Netherlands). The brain from the patient who donated her body to the Department of Anatomy was removed from the skull before scanning. The brains from the patients from the Department of Pathology were scanned surrounded by the skull. Non-contrasted thin slice reconstructions (0.8–1.0 mm) were analysed for hippocampal calcifications in different reconstructions, axial, coronal, and sagittal in the brain window setting (Center: 40 Hounsfield Units, Width: 80 Hounsfield Units) using the Philips IntelliSpace Portal 7.0 (Philips Healthcare, Best, The Netherlands). Calcifications were bilaterally scored on severity as absent, mild (one dot), moderate (multiple dots) or severe (confluent) (Fig 1) as described first by Kockelkoren *et al*. [8]. Calcifications on the CT scans are seen as a group of white voxels with a density similar to bone (Fig 1). The hippocampi were scored by an experienced observer. His agreement in comparison with other observers was previously investigated (kappa 0.80) [8]. The observer was blinded to the histological results.

### Microscopy—Histological study of nine hippocampi of seven patients

In two patients (patient number 1 and 2, corresponding with Tables 1 and 2), both hippocampi (hippocampus number 1 to 4, corresponding with Table 2) were evaluated by histology, in five patients (patient 3 to 7, corresponding with Table 2) one hippocampus was evaluated. The tail, body and head of the hippocampus were sampled in four of the nine hippocampi (hippocampus number 1 to 4). In the other five hippocampi, only the body was evaluated (due to the standard procedure of the Department of Pathology, in which the diagnosis of the disease the patient suffered from is the most important purpose. For each patient 22 standard pieces of the brain were cut out and three pieces with own content, these three extra pieces for diagnosis of the disease were more important than pieces for research purposes. This is why not always the head, body and tail of the hippocampus were analysed). Samples of the brains were put into cassettes. All cassettes were dehydrated in a graded series of ethanol (up to 95%) and embedded in paraffin. Subsequently, the paraffin embedded samples were cut into slices of 6 μm and put on glass microscope slides for pathological study. All sections were stained with hematoxylin and eosin (HE) for characterization of the cytoarchitecture [10]. In addition to this, the hippocampal sections were stained with von Kossa method for detection of calcium [10] and Elastica van Gieson stain to identify elastic connective tissue in vessel walls [11]. An experienced neuropathologist analysed all the specimens. The pathologist was blinded to the results of the CT scan. The calcifications in histology were quantified by the amount of vessels that were positive for calcium. Less than five vessels affected with calcium was considered mild, more than five non-confluent calcified vessels affected was considered moderate and more than five confluent calcified vessels that often had large calcium beads was considered severe.

**Table 2:** Overview of hippocampal calcifications on CT scan and in histological study.

Patient number	Hippocampus number	CT scan	Histology
		Severity	Severity
**1**	**1**	++	+++
	**2**	++	+
**2**	**3**	+++	+++
	**4**	+++	+++
**3**	**5**	+++	+++
**4**	**6**	+	+
**5**	**7**	-	-
**6**	**8**	-	-
**7**	**9**	-	-

The patient numbers correspond with the patients presented in Table 1.

Severity: whether the calcification is severe +++, moderate ++, mild + or absent -.

CT scoring: absent, mild (one dot), moderate (multiple dots) or severe (confluent) [8].

Histology scoring: absent, mild (less than five vessels affected with calcium), moderate (more than five non-confluent calcified vessels) or severe (more than five confluent calcified vessels that often had large calcium beads).

### Macroscopy of the brain of one patient

**Fig 2:**
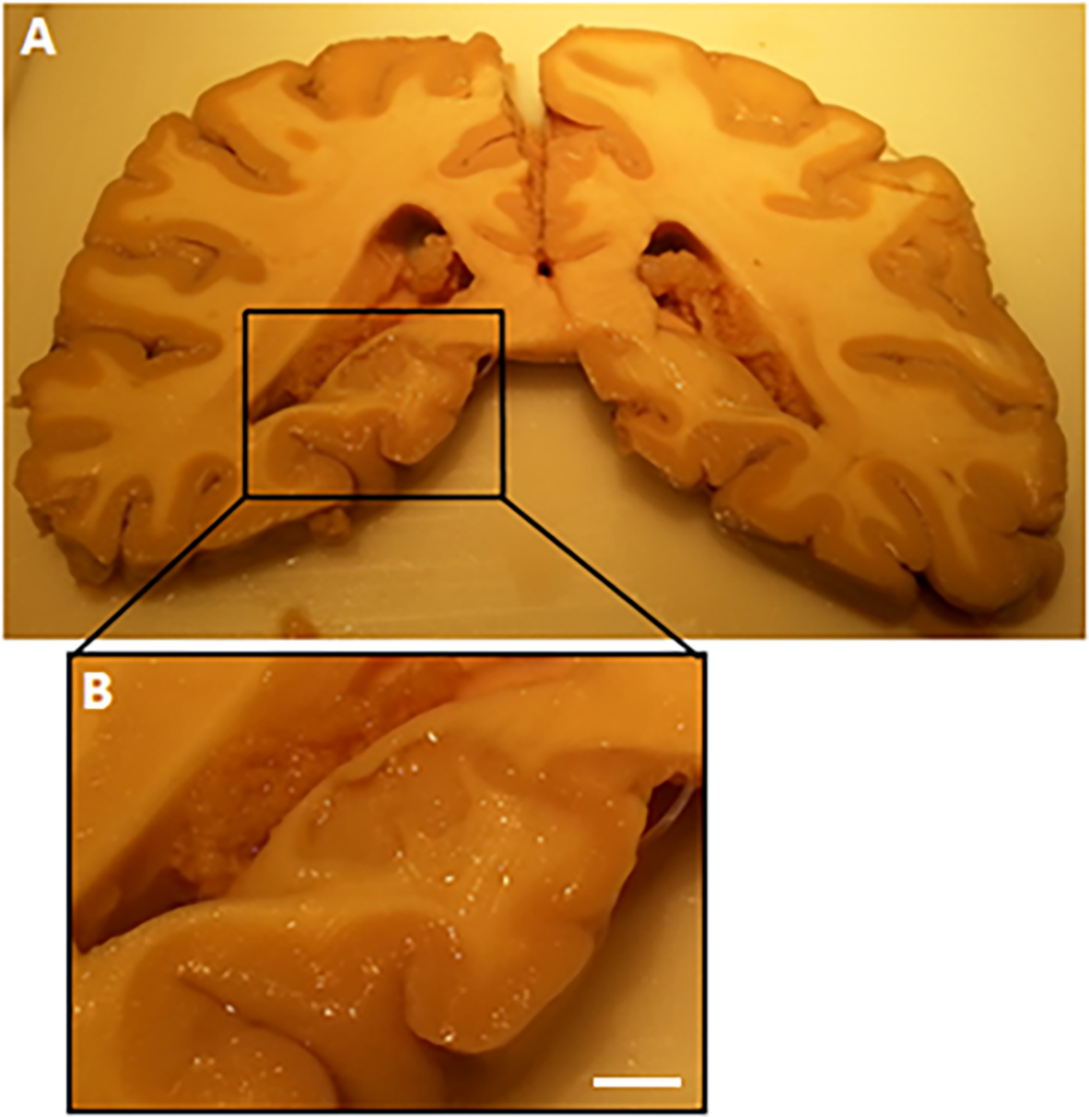
Coronal brain slice after dissection of the brain (of patient number 1). (A) Coronal slice with the posterior part of the hippocampus (tail). (B) Close up of the posterior part of the hippocampus (scale bar = 10 mm). Choroid plexus is visible in the lateral ventricle. The close up shows the sample for further histological staining.

In one patient (patient number 1, as described in Tables 1 and 2), a highly detailed analysis of the whole brain and both hippocampi was performed, to find more detailed information about the location of the calcifications in the vessel structures. This particular brain was obtained from the Department of Anatomy and had been fixed in 3% buffered formaldehyde. The brain was cut into coronal slices of 10 mm thickness, 25 samples were taken and put into cassettes. Fig 2 shows a coronal slice of the posterior part of the brain. The hippocampus as seen in Fig 2B was removed into a cassette and used for histological study. The important samples are bilateral hippocampus anterior (head), middle (body), and posterior (tail), beside the other samples for general microscopy.

## Results

### Computed tomography

Calcification of the hippocampus was found bilaterally in six of the nine hippocampi on CT scan. Three hippocampi had severe calcifications, two had moderate calcifications, one had only mild calcification and three had no calcifications. Fig 1 shows, as example, the routine CT images in different planes with hippocampal calcification.

### Correlation of CT findings with histology

In all six hippocampi with calcifications and all three hippocampi without calcifications on CT, these results from CT were confirmed by histology. Of the six calcified hippocampi three were considered as severe calcifications, two as moderate and one as mild. The correlation between calcifications on CT and the histopathological findings is shown in Table 2. Validated with histological staining, four subjects had severe calcifications, two had mild calcifications, and three subjects had no calcifications.

### Vascular localization of hippocampal calcification

We analysed one brain with two hippocampi (patient number 1, as described in Tables 1 and 2) in more detail than the other specimens. To indicate the exact location of the calcifications in the vessel structures.

In the anterior part, the head of the hippocampus, no calcification was noticed, neither in the left nor in the right hippocampus. The middle part, the body of the left hippocampus, showed mild calcification localized in the precapillaries and capillaries in the molecular layer of the dentate gyrus (DG) and Cornu Ammonis 1 (CA1).

**Fig 3:**
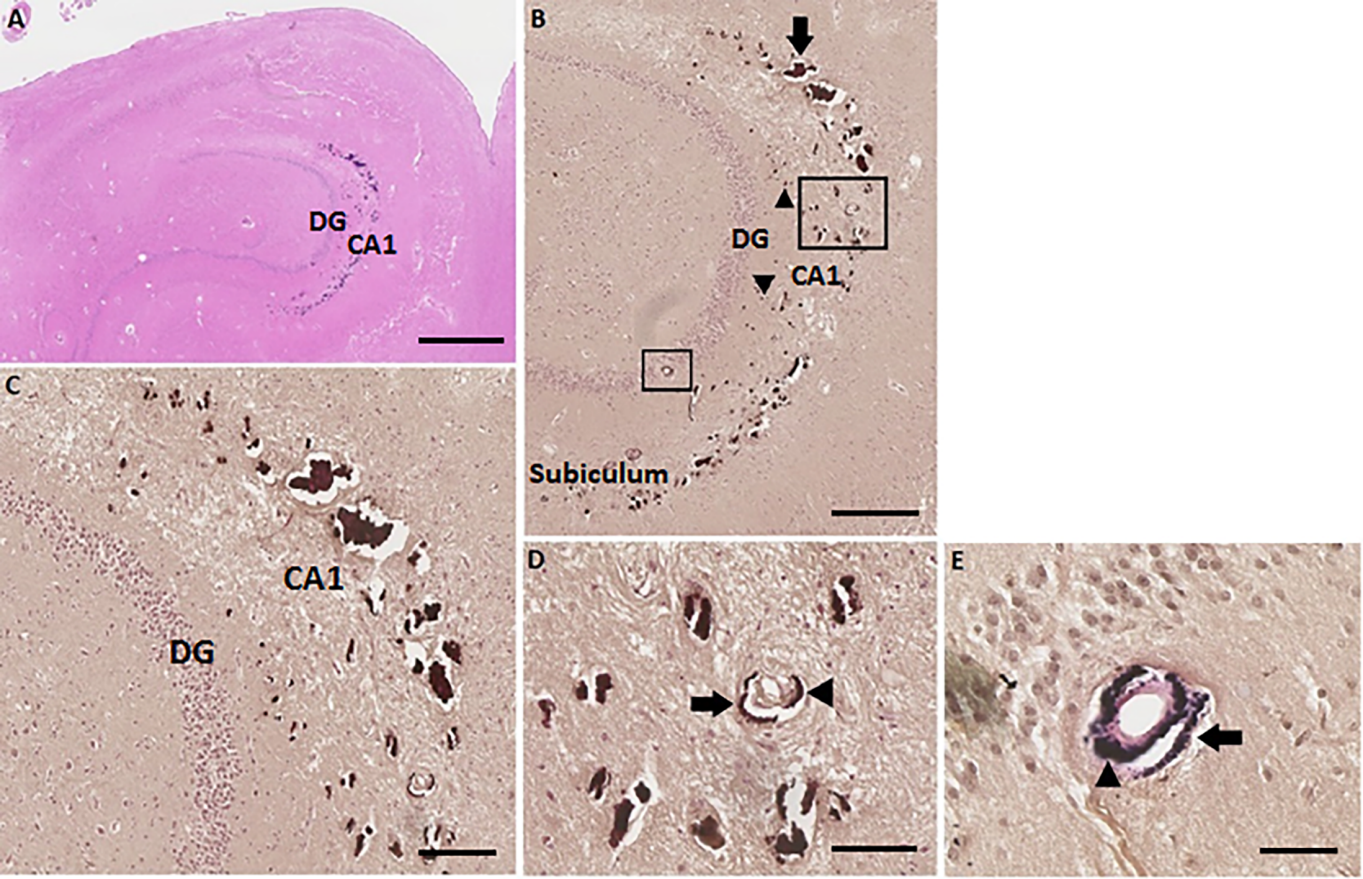
Severe calcifications in the tail of the left hippocampus (hippocampus number 1) of patient number 1 (as described in Tables 1 and 2). (A) HE stain overview of the left hippocampal tail. Scale bar = 1 mm, 4x magnification. (B) Von Kossa-positive deposits in precapillaries and capillaries (arrowheads) in the molecular layer of the DG and CA1. Calcifications of the bigger vessels, mostly arteria (arrow), in the molecular layer of CA1 are clearly observed. Two arteria which are calcified are surrounded, one in the molecular layer of the CA1 and one in the granular layer of the DG. The calcifications spread out into the molecular layer of the subiculum. Scale bar = 600 μm, 10x magnification. (C) Zoomed in on the big calcifications of precapillaries, capillaries and bigger vessels in the molecular layer of the DG and CA1. Epithelial cells of the vascular wall are not seen, because of the big calcification deposits. Scale bar = 400 μm, 20x magnification. (D) Enlarged image of a von Kossa-positive calcified artery in the molecular layer of the CA1. Calcification of the tunica adventitia (arrow) and the tunica media (arrowhead) are identified. Scale bar = 200 μm, 40x magnification. (E) Calcified artery in the granular layer of the DG shown with Elastica van Gieson stain. Calcification of the tunica adventitia (arrow) and the tunica media (arrowhead) are identified. Scale bar = 100 μm, 40x magnification.

The posterior part, the tail of the left hippocampus, showed severe calcification of precapillaries, capillaries and the arteries (Fig 3). These calcification beads, shown in Fig 3C, were larger in comparison to the mild calcification observed in the left hippocampal body. Also, an increase of number of calcifications was noticed. In this severe stage, calcifications were localized in the granular layer of the DG and the molecular layer of the DG and CA1 and spread out over the border of CA1 into the molecular layer of the subiculum (Fig 3B). Fig 3D shows a calcified artery in the molecular layer of the CA1. The Elastica van Gieson stain clearly showed the structure of an artery, with calcification in the tunica adventitia and the tunica media, as seen in Fig 3E. In some slices, it was difficult to distinguish the structure of the vessels in this severe stage of calcification, because sometimes no vessel walls were observed, only the calcium deposits.

**Fig 4:**
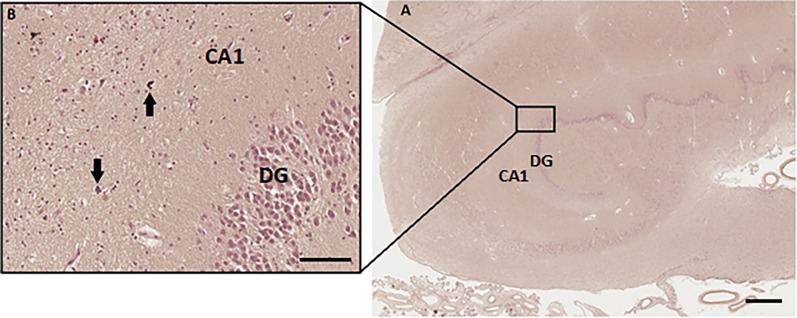
Calcification of precapillaries and capillaries in the right hippocampal tail (hippocampus number 2) of patient number 1 (as described in Tables 1 and 2). (A) Overview of the right hippocampal tail. Scale bar = 1 mm, 4x magnification. (B) Zoomed picture of von Kossa-positive stain, calcifications are located in the precapillaries and capillaries (arrows) in the right hippocampal tail. Scale bar = 100 μm, 40x magnification.

The right hippocampus contained no calcification in the body. The posterior part, the tail of the right hippocampus, showed mild calcification in precapillaries and capillaries (Fig 4).

### Onset of calcification

In the two brains (patient number 1 and 2, as described in Tables 1 and 2) of which the tail, body and head of both hippocampi were analysed, a striking pattern was seen. In one brain, both hippocampi were severely calcified in the tail, moderately calcified in the body and mildly calcified in the head. In the other brain, the tail of one hippocampus was affected by severe calcification and the body of that hippocampus showed mild calcification. In the other hippocampus only the tail was mildly calcified. This calcification pattern indicates that the calcification first appears in the tail, followed by the body, and subsequently when severe also occurs in the head of the hippocampus. This remains speculative and needs further confirmation.

### Choroid plexus calcification

**Fig 5:**
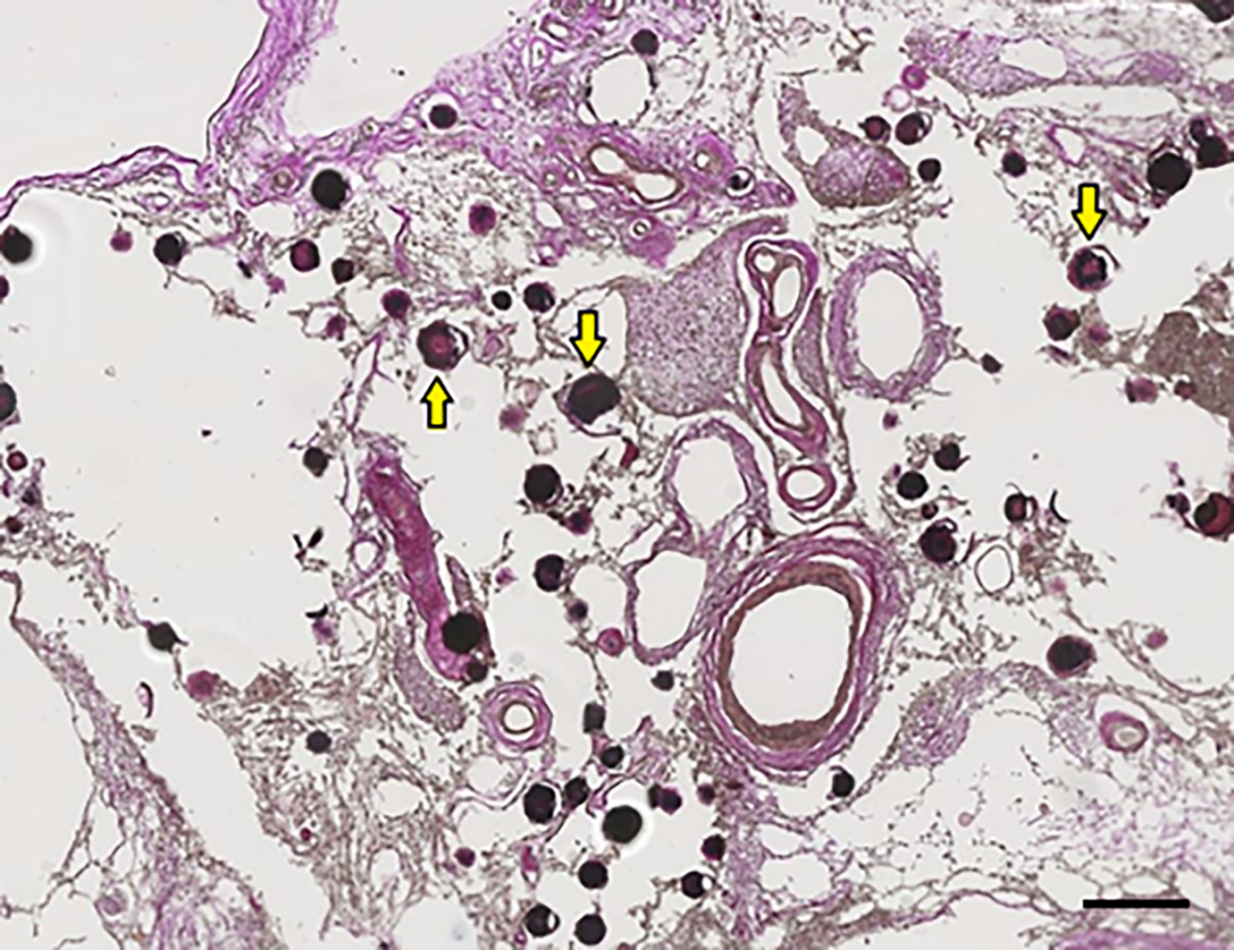
Calcification of the choroid plexus on the left side of patient number 1. The dark red/black dots, some are indicated with yellow arrows, are calcifications of precapillaries and capillaries in the choroid plexus on the left side of the brain. Scale bar = 1 mm, 20x magnification.

To demonstrate the difference between hippocampal calcification and calcification of the choroid plexus, a picture of choroid plexus calcification is included (Fig 5). Small vessels, precapillaries and capillaries were calcified in the choroid plexus. The large arteries and veins were less calcified.

## Discussion

The main finding of this study is that hippocampal calcifications as observed on CT images are confirmed with histology to be located in the hippocampal vasculature. Mild calcifications were found posterior in the tail of the hippocampus and extended into the body, toward the head of the hippocampus in severe stages. Whether this is a pattern that repeatedly will occur in different patients, should be further investigated and confirmed. The calcifications started in the precapillaries and capillaries of the molecular layer of the DG and CA1. Severe calcification expands in the molecular layer of the DG and CA1, the granular layer of the DG and over the border of CA1 to the molecular layer of the subiculum, as observed in four severe calcified hippocampi. The vascular calcifications seemed to be located in the adventitial and medial layer of the vessel wall. Our study is the first study that confirmed the vascular origin of the hippocampal calcifications that are detectable on CT.

Limited research has been done on calcifications of the hippocampus. A histopathological study from 2002 described vascular fibrosis and calcification in the hippocampus in subjects with Alzheimer’s disease (in 59% of the patients), Down syndrome patients (in 4%) and control aging patients (in 40%) [10]. In that study, just as in ours, calcifications were found in precapillaries and capillaries of the molecular layer of the DG and expanded to the granular layer and polymorphic layer of the DG, and to the molecular layer of the CA1. These calcifications found in the study of Wegiel *et al*. [10] apparently start in the hippocampal tail and spread in severe stages to the body and in some cases to the head of the hippocampus. A finding that we have seen as well in two brains of which the tail, body and head of the four hippocampi were analysed. This pattern remains speculative and needs further confirmation. In the study of Wegiel *et al*. [10] was found that the calcifications caused changes of the endothelial cells in the wall and lumen of vessels. In later stadia, the vascular wall became thicker and the calcified beads increased, these calcium deposits degraded the vessels and occlusion of blood circulation was noticed. Which indicates that an early diagnosis of these calcifications, with for example CT, will be valuable. Also in our study we observed degraded vessel walls and big calcium deposits, which might be implicated in the pathophysiology of vascular degradation. Our study demonstrated, through comparison with histological staining, that the calcifications found on CT images are indeed located in the hippocampal vasculature. The hippocampal calcifications are located in the molecular layer of the DG and CA1 and in the granular layer of the DG as observed in all analysed hippocampi positive for hippocampal calcifications. Interestingly, these locations correspond with the location of vascular fibrosis and calcifications described in the histopathological study of Wegiel *et al*. [10].

Calcifications can be detected by neuroimaging, and are seen as high density areas on CT scans, while these calcification areas are considerable less visible on magnetic resonance imaging (MRI) [12]. Indicating that CT will provide a better diagnosis for hippocampal calcifications than MRI. A second study from 2012 [9] was the first study to describe hippocampal calcifications on CT images. Due to thin slice CT images, a distinction between choroid plexus calcifications and hippocampal calcifications could be made. In total, 300 randomly selected CT scans were analysed, of which intrahippocampal calcification was demonstrated in 47 patients, all these patients were older than 50 years of age. The authors concluded that intrahippocampal calcification appeared with increasing age [9]. With our study we demonstrated with histology that the CT detected calcifications were indeed located in the hippocampus.

The effect of hippocampal calcifications on cognitive functioning was investigated in a recent pilot study from 2016 [8]. Kockelkoren *et al*. [8] examined memory clinic patients and controls. In this study, it was found that hippocampal calcifications were three times as prevalent in patients of the memory clinic compared to control patients. Furthermore, memory clinic patients with hippocampal calcifications showed lower cognitive functioning measured with Mini Mental State Exam. In two other smaller studies no significant difference in the presence of hippocampal calcifications between controls and a group of Alzheimer’s disease patients was found [10; 13]. More research should be performed to possibly confirm the correlation of hippocampal calcifications and dementia. In this study of Kockelkoren and colleagues it was hypothesised that the hippocampal calcification as seen on CT scan could be caused by vascular fibrosis and calcification, because it seems like it is located in the same region of the hippocampus [8]. Until now, this remained speculative, but our study provides support for this hypothesis by correlating CT findings to histology in the same brain.

In the Alzheimer’s disease patients in the study of Wegiel *et al*. [10], neuronal cell loss was found in the CA1 region and subiculum proper, comparable to hippocampal sclerosis [10]. This was suggested to be characteristic for Alzheimer’s disease patients. In normal aging patients, neuronal cell loss was rarely seen in the CA1 region of the hippocampus [12]. In our study we used material of normal aging patients. In histology we observed that the patients had some signs of aging, like amyloid-beta senile plaques, which was age-appropriate. We did not find obvious neuronal cell loss, which may be due to the fact that our subjects did not have Alzheimer’s disease [14].

The posterior cerebral artery supplies the hippocampus of blood. According to the flow territories, the branches of the hippocampal arteries can be divided into two groups. Blood supply to the body and tail of the hippocampus is from the middle and the posterior hippocampal arteries. The anterior hippocampal arteries supply the head of the hippocampus and the uncus [15]. Also the choroid plexus of the lateral ventricle is supplied by branches of the posterior cerebral artery [16]. Since the calcification in the vasculature in both the hippocampus and the choroid plexus is similarly located in the precapillary and capillary vessels, and both types of calcification have the same blood supply from the posterior cerebral artery, future research could be directed to a possible relation between these calcifications.

Future research may also focus on the effects of hippocampal calcifications on cognitive functioning. The main function of the hippocampus is learning and memory. Nowadays it is thought that the hippocampal sub regions, anterior (head) and posterior (tail), are involved in different aspects of memory. The exact distribution is still not known and different studies suggest different functions [17, 18]. Because the hippocampal tail is affected most early in this calcinosis process, it would be interesting to investigate the function of the posterior hippocampus in relationship with hippocampal calcifications. A potential link with cognitive decline and dementia could be investigated not only in memory clinic patients, but also in normal aging patients. In the limited medical history of our patients, we found that some patients had neurological damage and others had cardiovascular problems, both of which may be associated with calcifications in the brain. In future research a possible association between cardiovascular diseases and hippocampal calcifications can be investigated.

This study, in which we validated hippocampal calcifications as observed on CT scan with histological staining, is a crucial step for future research to investigate the underlying mechanism and consequences of these hippocampal calcifications. In the future we could detect the hippocampal vascular calcifications in early stages with thin slice CT scans. Thin slice CT scans could become a prognostic marker for cognitive decline [8] or other not yet investigated consequences of hippocampal calcifications. When finally the mechanism underlying hippocampal calcifications is known and this is related to a negative function for the patients, treatments on this mechanism could be investigated. With the prognostic marker of CT scans and applicable treatments, patients can be helped earlier.

Limitations of the current study are the lack of information about cognitive ability of the patients. Nothing can be said about a possible correlation between hippocampal calcifications and cognitive functioning of these patients. Secondly, the sample size of this study was relatively small, however, it is in our opinion sufficient to validate the calcifications on CT scans with histology, the main focus of this research. In the histology of four hippocampi we saw a possible pattern of calcification severity that expanded from tail toward the head of the hippocampus, although this is a limited number, the pattern was also observed by Wegiel *et al*. [10]. We think further studies with larger cohorts who underwent CT scanning can more firmly investigate this pattern and we did not specifically investigate this in the present study. A last limitation is the way we quantified the histological findings. There is not yet a clear standard for the quantification of hippocampal calcification in histology as it is the case for hippocampal calcifications on CT scan [8]. Therefore, we made an own quantification method for hippocampal calcification for histology.

In conclusion, we showed that all hippocampal calcifications observed on thin slice CT images are confirmed by histological staining to be of vascular origin. The calcifications in mild stage were found in precapillaries and capillaries of the molecular layer of the DG and CA1. In a more severe stage also the arteries in the molecular layer of the CA1 and the granular layer of the DG were affected. Calcifications were most often found in the hippocampal tail and in a more severe stage they also became visible in the hippocampal body and sometimes in the hippocampal head. In further studies, the consequences of these hippocampal calcifications on cognitive impairment or possible other functions of the hippocampus, can be investigated.
